# The Physicians Visiting List for 1878

**Published:** 1877-10

**Authors:** 


*The Physicians Visiting List for* 1878.—Lindsay and Blackiston, Publishers, Philadelphia.

As an evidence of the popularity of this List, it is only necessary to say that this is the twenty-seventh edition. It is not only useful to the medical practitioner, but also to the dental as an appointment book.

